# Co-evolution of vaginal microbiome and cervical cancer

**DOI:** 10.1186/s12967-024-05265-w

**Published:** 2024-06-11

**Authors:** Menglu Hu, Wentao Yang, Ruiyi Yan, Jiayu Chi, Qi Xia, Yilin Yang, Yinhan Wang, Lejia Sun, Ping Li

**Affiliations:** 1 Department of Radiotherapy and Oncology, Affiliated Kunshan Hospital of Jiangsu University, Kunshan, China; 2 School of Medicine, Southeast University, Nanjing, China; 3 Chinese Academy of Medical Sciences Peking Union Medical College, Beijing, 100730 China; 4 The First Affiliated Hospital, Nanjing Medical University, Nanjing, Jiangsu 210029 China

**Keywords:** Cervical cancer, Single nucleotide variation, Vaginal microbiome, Co-evolution

## Abstract

**Background:**

Exploration of adaptive evolutionary changes at the genetic level in vaginal microbial communities during different stages of cervical cancer remains limited. This study aimed to elucidate the mutational profile of the vaginal microbiota throughout the progression of cervical disease and subsequently establish diagnostic models.

**Methods:**

This study utilized a metagenomic dataset consisting of 151 subjects classified into four categories: invasive cervical cancer (CC) (*n* = 42), cervical intraepithelial neoplasia (CIN) (*n* = 43), HPV-infected (HPVi) patients without cervical lesions (*n* = 34), and healthy controls (*n* = 32). The analysis focused on changes in microbiome abundance and extracted information on genetic variation. Consequently, comprehensive multimodal microbial signatures associated with CC, encompassing taxonomic alterations, mutation signatures, and enriched metabolic functional pathways, were identified. Diagnostic models for predicting CC were established considering gene characteristics based on single nucleotide variants (SNVs).

**Results:**

In this study, we screened and analyzed the abundances of 18 key microbial strains during CC progression. Additionally, 71,6358 non-redundant mutations were identified, predominantly consisting of SNVs that were further annotated into 25,773 genes. Altered abundances of SNVs and mutation types were observed across the four groups. Specifically, there were 9847 SNVs in the HPV-infected group and 14,892 in the CC group. Furthermore, two distinct mutation signatures corresponding to the benign and malignant groups were identified. The enriched metabolic pathways showed limited similarity with only two overlapping pathways among the four groups. HPVi patients exhibited active nucleotide biosynthesis, whereas patients with CC demonstrated a significantly higher abundance of signaling and cellular-associated protein families. In contrast, healthy controls showed a distinct enrichment in sugar metabolism. Moreover, biomarkers based on microbial SNV abundance displayed stronger diagnostic capability (cc.AUC = 0.87) than the species-level biomarkers (cc.AUC = 0.78). Ultimately, the integration of multimodal biomarkers demonstrated optimal performance for accurately identifying different cervical statuses (cc.AUC = 0.86), with an acceptable performance (AUC = 0.79) in the external testing set.

**Conclusions:**

The vaginal microbiome exhibits specific SNV evolution in conjunction with the progression of CC, and serves as a specific biomarker for distinguishing between different statuses of cervical disease.

**Supplementary Information:**

The online version contains supplementary material available at 10.1186/s12967-024-05265-w.

## Background

Cervical cancer (CC) is a global public health problem, with an estimated 604,000 new cases and 342,000 deaths worldwide in 2020, according to the latest Globocan report [1]. Although vaccines have been developed, CC continues to be a major public health problem affecting middle-aged women, particularly in less-resourced countries [2]. Persistent human papillomavirus (HPV) infection is the main trigger for the development of CC. Currently, more than 200 different HPV types have been described to infect epithelial cells, which are further divided into low- and high-risk HPV types, depending on their carcinogenic potential [3]. Although HPV infection is a risk factor for the progression of cervical lesions and the development of CC, only a small number of women develop precancerous cervical dysplasia, with the highest risk of progression to cancer. Therefore, it is crucial to study other factors in the local cervicovaginal microenvironment that facilitate the transition from HPV infection to cervical precancerous condition.

Rapidly evolving evidence highlight the association between the vaginal microbiome and the acquisition of HPV, as well as the occurrence of cervical intraepithelial neoplasia (CIN) and CC [4, 5]. Multiple cross-sectional studies in various racial and ethnic cohorts have demonstrated that HPV-infected women exhibit a more diverse, *non-Lactobacillus* dominant vaginal microbiota [6, 7]. Furthermore, it has been found that increasing severity of CIN was associated with higher vaginal microbiome diversity and decreased relative abundance of *Lactobacillus spp* [8]. Thus, a vaginal microbiome dominated by a variety of *Lactobacillus* species is considered healthy. Additionally, anaerobic bacteria such as *Sneathia spp.* were significantly enriched in CIN samples in multiple studies [9] and were strongly associated with changes in immune mediators. Several recent reviews have shown that a dysbiotic vaginal microbiome plays a significant role in the development and progression of cervical diseases, including HPV infection (HPVi), CIN, and CC [10, 11]. Accordingly, numerous biomarkers based on changes in bacterial composition have been considered as potential biomarkers for HPVi and CC [12, 13].

Evidence indicate that notable evolution of the microbiome may occur prior to changes in composition and abundance during the progression of the host disease [14]. Theoretically, evolution depends on genetic variation. In-depth profiling of genetic variation can detect strain-level variations, such as single nucleotide variants (SNVs), short insertions/deletions, structural variants, copy number variations, and simple sequence repeats [14, 15]. Based on the gut microbiome, SNVs have been used as markers to distinguish subjects with colorectal cancer from healthy subjects [16].

Regarding the genital tract microbiome, population nucleotide diversity, selection metrics, and antibiotic resistance potential are associated with preterm birth [17]. This study offers a unique population genetic elucidation of the association between the vaginal microbiome and alterations in the host environment, implying that microbial genomes have also undergone co-evolution with human disease states. To date, no study has reported an association between vaginal microbiota and disease at the level of structural variations. Therefore, we hypothesized that specific genomic variations in vaginal bacteria correspond to the occurrence and malignant transformation of cervical diseases.

**Fig. 1:**
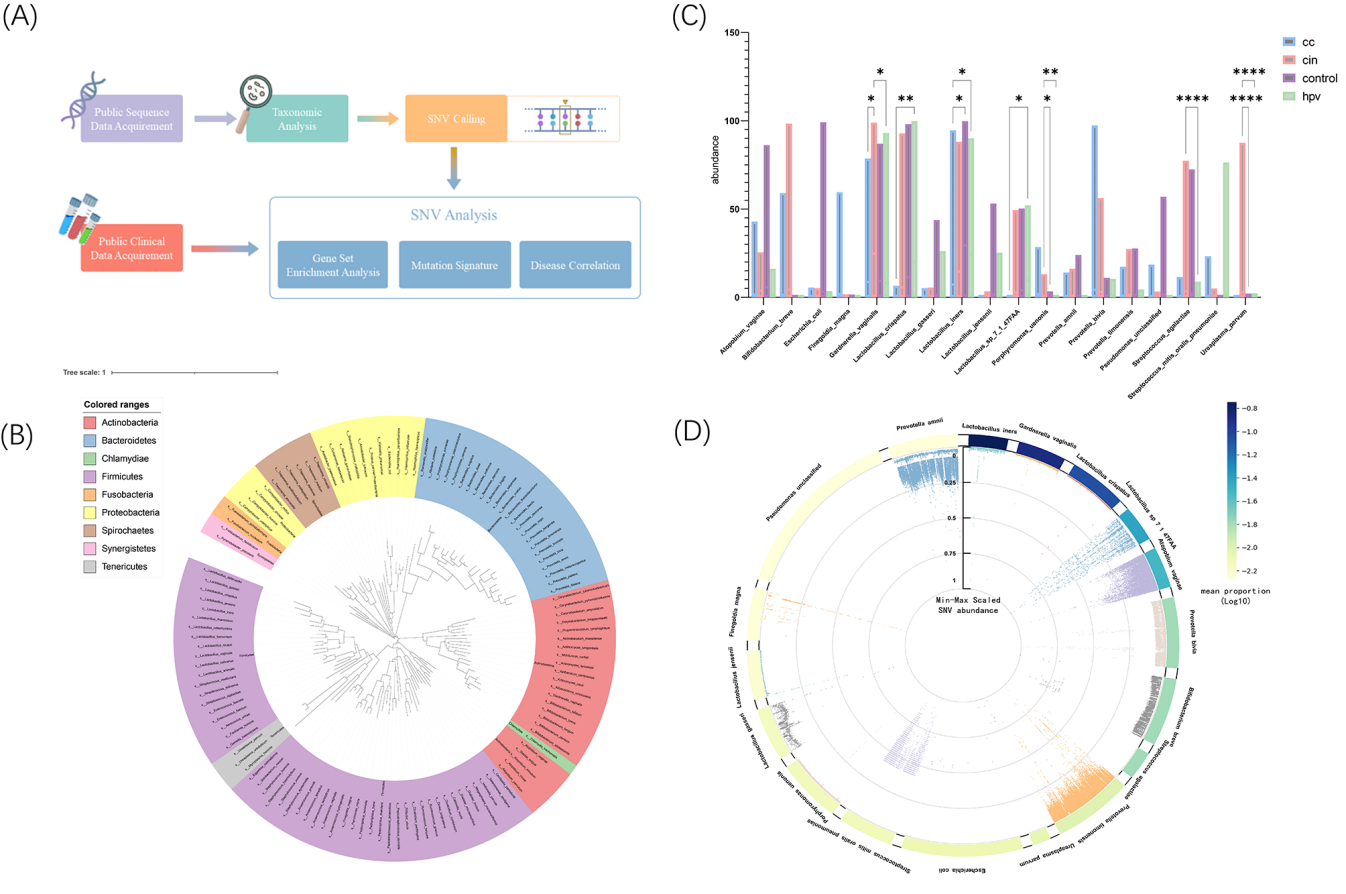
Experimental design and the integrated analysis of cervical cancer-associated microbiome. (**A**) Experimental design. A total of 151 human subjects with vaginal metagenomic data were downloaded from NCBI, comprising 42 cervical cancer patients, 43 cervical intraepithelial neoplasia patients, 34 HPV-infected patients and 32 healthy controls. Using MetaPhlan2, the relative abundance of every species in a single sample were calculated. Those species with an average relative abundance greater than 0.5% were selected for further analysis and single nucleotide variant (SNV) calling. All identified SNVs were annotated to their corresponding genes based on their genomic locations. Mutational patterns and signatures were extracted using base substitutions and additionally included information on the sequence context. Subsequently, the top 500 genes exhibiting the highest number of SNVs within each group were selected and annotated to Kyoto Encyclopedia of Genes and Genomes (KEGG) metabolic pathways for assessing functional alterations. Finally, diagnostic models incorporating these multimodal microbial markers and the random forest algorithm was built. (**B**) Evolutionary tree showing MetaPhlan2 extracts for all species. (**C**) Abundance of 18 strains selected according to abundance and breadth in different subgroups. (**D**) Overall screening of and demonstrated that a total of 71,6358 non-redundant mutations were annotated into 25,773 genes belonging to 427 contigs, and the species abundance of the mutations belongs to are also shown

The primary objective of this study was to investigate the coevolutionary microbial signatures associated with the progression of CC, encompassing taxonomic, functional, and SNV levels (Fig. 1A). Analyses incorporating enriched pathways and their associated genes may provide insights into the underlying mechanisms by which alterations in the bacterial landscape contribute to the development of CC. The potential utility of microbial signatures as effective diagnostic biomarkers has also been assessed. Consequently, this study expands the understanding of genetic alterations in vaginal microbial communities and offers an unprecedented opportunity for early detection of noninvasive CC.

## Method

### Sequence and clinical data source

The vaginal shotgun metagenomic sequence data of internal training and validating set was downloaded from NCBI PRJNA771720 (https://www.ncbi.nlm.nih.gov/sra/?term=PRJNA771720), which was uploaded by Liu, H. et al. [18]. A total of 151 samples’ paired-end reads were acquired including 42 individuals with CC, 43 individuals with CIN, 34 HPV carriers without CC and CIN, and 32 healthy individuals. Besides, in order to further illustrate the performance of our final diagnose model, two external testing sets was downloaded: NCBI PRJNA1057216 (https://www.ncbi.nlm.nih.gov/sra/?term=PRJNA1057216) and NCBI PRJNA779415 (https://www.ncbi.nlm.nih.gov/sra/?term=PRJNA779415). PRJNA1057216 contains samples from 54 patients with CC or CIN, while PRJNA779415 includes 47 samples from individuals without CC and CIN and was uploaded by France MT et al. [19].

### Identification of microbial species, SNV calling and SNV biomarkers generation

With the implement of MetaPhlan2 [20], the original reads were directly annotated to a pre-built microbial genomics database and the relative abundance of every species in single sample were calculated. Those species with an average relative abundance greater than 0.5% were selected for the next SNV calling and their genomes and annotation profiles were downloaded from NCBI (see Table S1). For SNV calling, the original metagenomics reads were mapped to the reference genomes to discover all SNVs’ sites and depths with Bowtie2(v1.1) [21], SAMtools(v1.1) and BCFtools(v1.8) [22]. Subsequently, all SNVs were annotated to the corresponding genes according to their sites using Python(v3.9.12). For each sample, the number of SNVs in each gene, contig were calculated and were defended as biomarker-gene and biomarker-contig, respectively. The depths of each SNV were also recorded and defined as biomarker-SNV. Additionally, the relative abundance of species in each sample are defended as biomarker-taxa. Notably, the value of each SNV-related biomarker was further divided both by the relative abundance of the species it belongs to and the base counts of shotgun metagenomic sequence data to amend the bias derived from sequencing and species abundance. The Manhattan plot showing the SNV spots and species abundance was plotted by R package “CMplot” (v4.3.1) [23].

### Functional enrichment analysis

The protein sequences of the selected reference species were downloaded from NCBI and were annotated to KEGG metabolic pathways by eggNOG-mapper (v2) [24]. For each cohort, the top 500 biomarker-gene with the largest number of SNVs were selected, respectively, and the matched proteins were recorded if exists, according to the annotation profiles from NCBI (see Table S1). The KEGG enrichment analysis was accomplished by TBtools(v1.111) [25]. For the intersection part of the enriched pathways both in different cohorts, Wilcoxon rank-sum test was employed in R software to identify the different levels of enrichment in two group. Finally, the relevant figures was plotted by R software and http://www.bioinformatics.com.cn, an online platform for data analysis and visualization.

### Mutation spectrum and mutation signature

SNPEFF [26]was used to annotate the mutations in vcf files of different samples. Firstly, a custom database was established according to the gff file of the reference bacteria, and the bin file was generated. Then, SNVs were annotated according to the vcf file, and generate a snp.eff.vcf file for analyzing mutation types. Next we use snpefftomaf.pl to map the SNPEff. The vcf file is converted to a maf file, and maftools [27] is used to create a waterfall chart showing the mutation frequency. For mutational signatures of bacteria, we derived 96 mutational Signatures of SNVs across different groups by make a new BSGENOME data package, followed by inferring somatic signatures from single nucleotide variant calls, providing a basis for follow-up studies with specific codes.

### Filter of biomarkers and model construction

Recursive feature elimination (RFE) was employed to screen the biomarkers respectively using R software., and the biomarkers of species abundance and the biomarkers of SNV in gene were sent in the final model as input.

Four random forest diagnose models were trained using 4 different types of biomarkers individually to evaluate their values in discriminating individuals from 4 groups. Then, a final diagnose model using filtered biomarkers was trained and tested in external testing set. The R package “randomForest” (v4.7-1.1) was applied for the construction of random forest models using the default hyperparameters. The models was trained using the 85%-15% train-test ratio and was evaluated with a fivefold cross-validation approach. The figure of RFE selection, chart of rank of Gini coefficient and the receiver operating characteristic curve were plotted by R package ggplot2 (v3.4.2) [28].

### Statistics statement

The statistical analyses were conducted using R software. Two-way repeated measures ANOVA followed by Bonferroni post-test was applied to the comparison of species abundance in different groups, the comparison of SNV counts belongs to different mutation type (missense, silent and nonsense) and the comparison of C > T/T > C SNV proportion in groups. Statistical analysis was performed using GraphPad Prism software. In the part of functional enrichment analysis, the pathways that enriched in multiple cohorts were further compared using the Wilcoxon rank-sum test. *P* values were considered significant at *P* < 0.05. Randomforest test was performed by the “randomForest” package.

## Result

### Characteristics of included cohort

In this study, the internal discovery samples belonging to project PRJNA771720 (*n* = 151) were included to evaluate vaginal microbiome changes as CC progressed (from control, HPV acquisition, to intraepithelial neoplasia, and cancer). The collection of all metagenomics sequences from cervical samples and associated clinical data was previously conducted by Liu, H. et al., and subsequently deposited in the NCBI Sequence Read Archive database [18]. In total, 151 samples were collected from 42 CC patients, 43 CIN patients, 34 HPVi patients, and 32 healthy controls. The external testing samples belonging to project PRJNA1057216 (*n* = 54, CC or CIN) and PRJNA779415 (*n* = 47, without CC and CIN) [19] were downloaded for the final diagnose model. From both the internal discovery and external testing cohorts, vaginal microbiota profiles were assessed using metagenomic shotgun sequencing to analyze microbial diversity across all taxonomic levels.

The internal discovery cohort consisted of cervical samples collected from patients with a Han Chinese background in Wuhan, China. The patients were stratified into disease and healthy control groups based on their HPV infection status. Within the disease group, individuals without cervical lesions were classified into the HPVi subgroup. Those categorized as having CIN and CC represented the first occurrence of these two diseases. The external testing cohort from PRJNA1057216 consists of 54 samples from patients with CC or CIN. As the further precise label is not available, these samples were then uniformly labeled as tumor group. The external testing cohort from PRJNA779415 contains 47 females without CC and CIN, who were then uniformly labeled as non-tumor group.

### Cervical cancer-associated microbial taxonomic alterations

Species annotation using MetaPhlan2 revealed 12 phyla, 21 classes, 35 orders, 64 families, 97 genera, and 202 species in the vagina (Fig. 1B). Consistent with previous studies, at the phylum level, *Firmicutes, Actinobacteria* and *Bacteroidetes* were dominant [29]. Unlike other anatomical sites, most vaginal communities were dominated by one or more species of *Lactobacillus* and *Enterococcus*, both of which belong to *Firmicutes*.

A genome library was constructed using the whole genomes of 18 strains with a sequencing depth of > 10×(Fig. 1C). These results suggest that patients with the presence of cervical lesions, have a lower proportion of *Lactobacillus* spp. than healthy controls. In addition, the predominance of *Bifidobacteriaceae breve*, with a minority of *Atopobium vaginae* and *L. crispatus* appears to be a risk factor for CC and CIN. These findings were consistent with previous research [30]. However, unlike previous studies, *Gardnerella vaginalis* was enriched in both HPVi and CIN patients but downregulated in CC patients (Fig. 1C). Furthermore, we conducted analysis on the SNVs in the aforementioned species and exhibited the correlation between species abundance and average relative abundance of SNVs. As depicted in the figure, no complete correspondence was observed between the average relative abundance of SNVs and species abundance. Notably, certain species with relatively low abundance, such as *Prevotella amnii* and *prevotella timonensis*,exhibited a high relative abundance of SNVs (Fig. 1D).

### Microbial gene alteration associated with CC progression

**Fig. 2:**
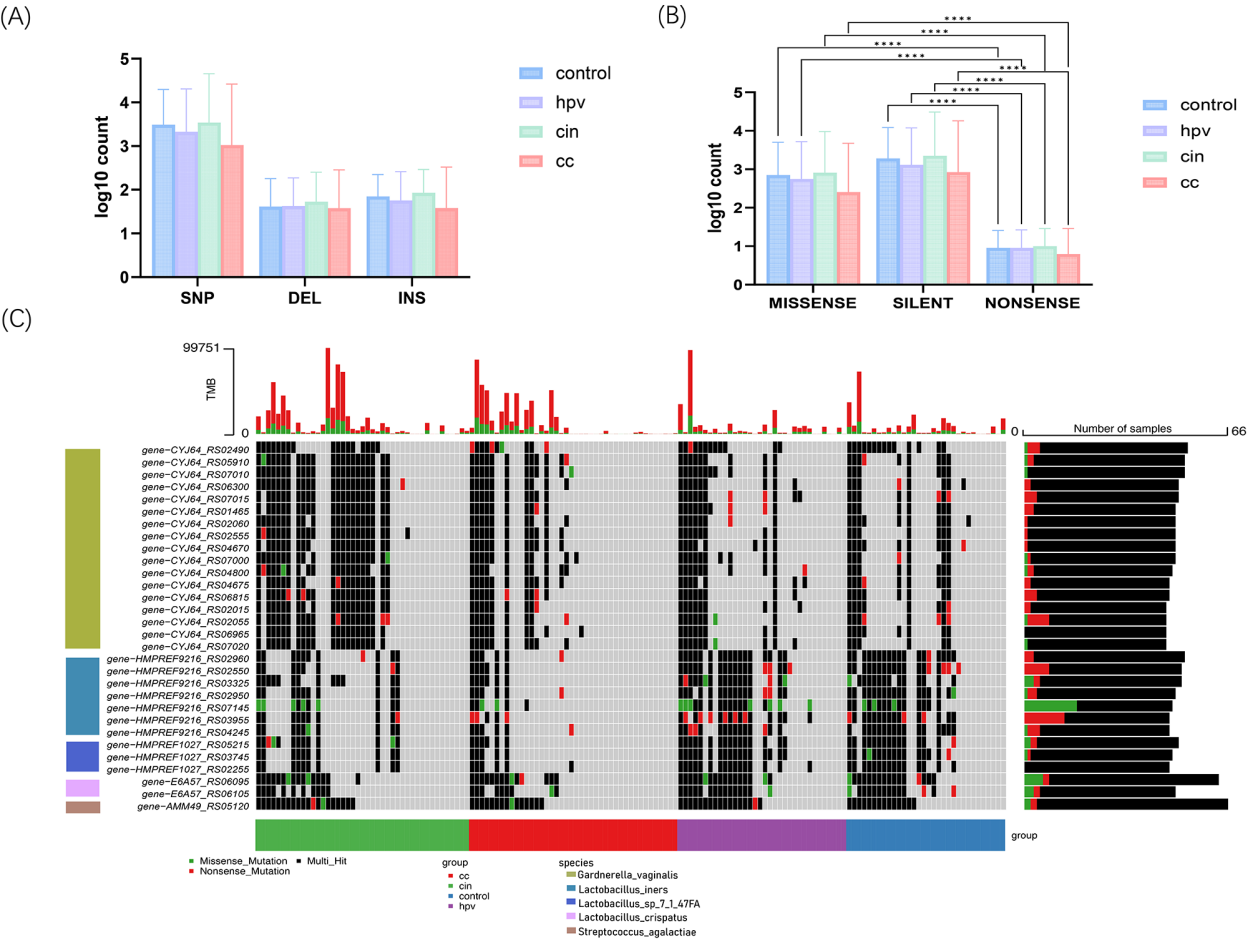
Microbial gene alteration among cervical cancer progression. (**A**) Comparison of the number of Snp\ins\del between the four groups, with the horizontal coordinates log transformed. (**B**) Proportions of silent, nonsense, and missense mutation types between the four groups. (**C**) Waterfall plot. Displays mutation types and frequencies in the top 30 genes with the highest mutation frequencies for CC, CIN, HPVi and control. Genes are classified according to the strain to which they belong. The horizontal coordinate represents samples and vertical coordinate represents genes. Different colors denotes different mutation types. The top bar reflects the proportion of different mutations within different samples, and the right bar reflects the proportion of total mutation types in all samples for the gene

Microbial genetic variations, including SNV, deletions, and insertions, represent potential alterations at the intra-species strain-level of microbial functionality (Fig. 2A). Thus, we characterized the mutation profiles of the vaginal microbiome during CC pathogenesis.

We examined the SNV signatures of different cervical statuses against 18 microbial strains (relative abundance > 0.5%) in the metagenomic dataset (Table. S1). Among all mutation types, the frequency of silent mutations demonstrated a higher overall trend compared to the other two mutations. However, regardless of being silent, nonsense, or missense mutations, similar mutation frequencies were observed among the four groups, respectively (Fig. 2B). A waterfall diagram identified the top 30 genes with the highest SNV counts, most of which were associated with multi-hit mutations, indicating their involvement in multiple types of genetic alterations. Among these genes, most mutations were observed in three *Gardnerella vaginalis*, along with common occurrences in *Lactobacillus spps* and.*Streptococcus agalactiae*, which are strains closely associated with vaginal dysbiosis (Fig. 2C). Specifically, mutations in genes within *Gardnerella vaginalis* exhibit a higher prevalence within the CC and CIN groups, whereas mutations in *Lactobacillus spps* and *Streptococcus agalactiae* are more prevalent among the remaining two groups.

Additionally, SNVs located in metabolism-related genes showed diverse mutation types across the four groups. Notably, the CYJ64-RS02490 gene displayed a higher likelihood of nonsense mutations, specifically in the CC group compared to other groups, suggesting a potentially lower expression level of the CadD family cadmium resistance transporter. In conclusion, these analyses underscore the importance of microbial genetic variations in the pathology of CC and highlight the potential diagnostic utility of SNVs.

### Microbial mutation signature alternation during evolution

**Fig. 3:**
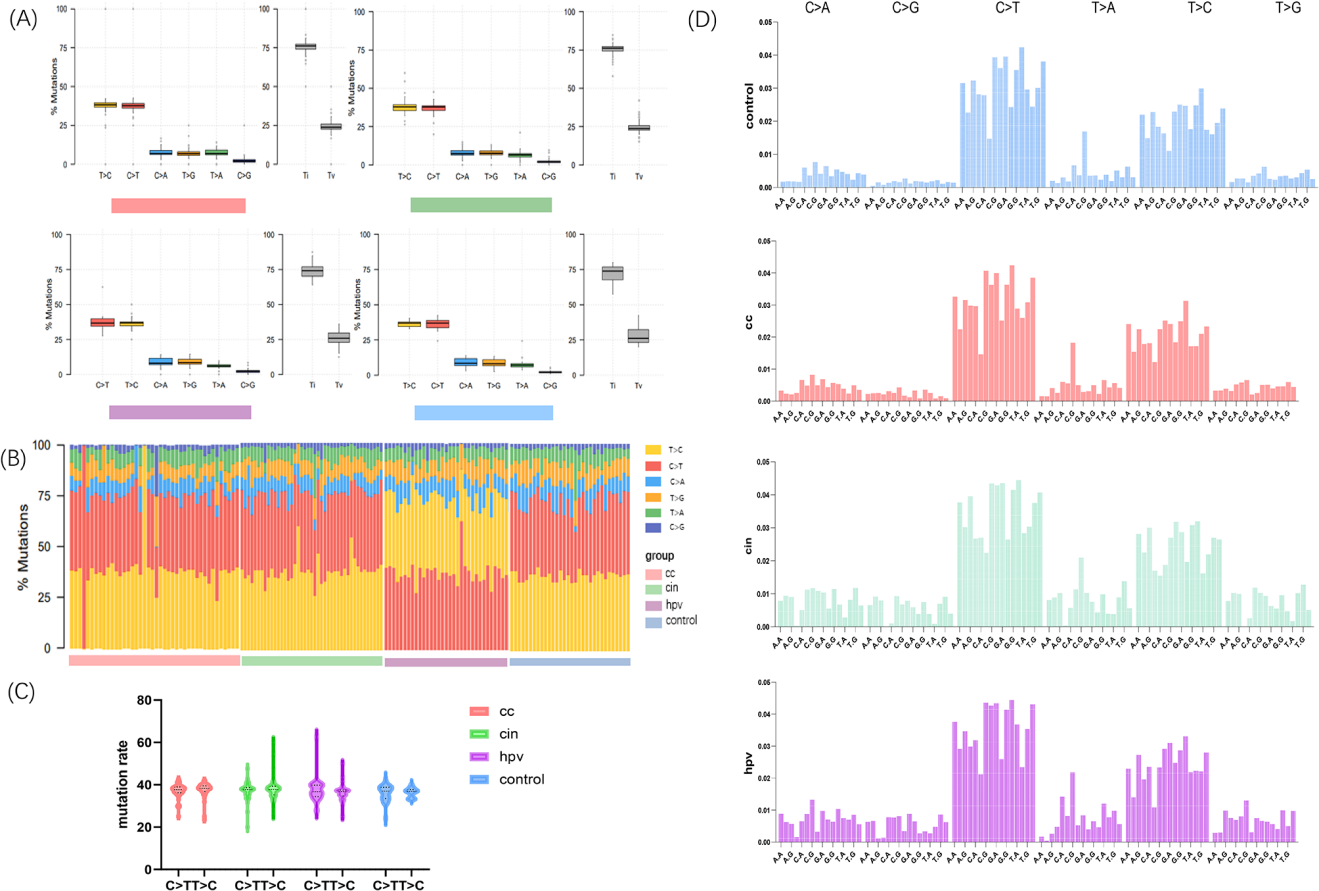
Microbial mutational patterns and signature alternation during evolution. (**A**) Comparison of mutational signatures based on base substitutions and Ti\Tv between the four groups. (**B**) Differential microbial mutational signature contribution associated with CC progression. (**C**) Abundance of snv with base mutation types C > T and T > C in CC, CIN, HPVi and control groups. (**D**) Characterization of 96 mutations in CC, CIN, HPVi and control groups based on the reference genome of bacteria. The base substitution at the mutation site contains six types: C > A, C > G, C > T, T > A, T > C and T > G. Four bases (A, T, C, G) can be paired on each side of the mutation site (5′ and 3′ ends), resulting in 96 possible mutation types (6 base substitution types at the mutation site × 4 5′ bases × 4 3′ bases). CC, cervical cancer; CIN, cervical intraepithelial neoplasia, HPVi, Human papilloma virus infection; Ti/Tv, the ratio of transitions to transversions for the pair of sequences

Somatic cells in various cancers exhibit distinct patterns of base replacement during development, which can be regarded as a mutational signature [31]. We investigated whether this signature was also evident in microbial SNVs. We extracted mutational signatures based on base substitutions and identified 12 distinct mutational possibilities. Based on the principle of complementary base pairing, only six classes were identified (C > A, C > G, C > T, T > A, T > C, T > G). Notably, the ratio of transitions to transversions for the pair of sequences (Ti/Tv) was 3. Specifically, the frequencies of T > C and C > T substitutions were the highest, whereas C > G exhibited the lowest frequency (Fig. 3A). When investigating the contribution of mutational signatures, we discovered that C > T was greater than T > C only in the HPVi group, while the rest of the groups were opposite (Fig. 3B and C, Table S2).

Additionally, information regarding the sequence context of each mutation has been incorporated. We integrated the data on the nucleotides immediately preceding and following each mutated base to classify the 96 bacterial mutation patterns (Fig. 3D). The mutation patterns in both the HPVi and CIN groups exhibited similarities, which was designated as signature (A) While the mutation pattern of CC group can be defined as signature (B) Both signatures A and B are characterized by a predominance of C > T substitutions at the NpCpG trinucleotides. However, a higher proportion of C > A, C > G, and T > G substitutions was observed in signature A than in signature B. Considering the progression of cervical lesions, the transition from signature A to B may indicate a significant shift in microbiome genetic signatures during malignant transformation.

Because there is currently no existing database for the 96-mutation signature of bacteria, we were unable to establish a correspondence with mapping. Hence, we used these data as reference cases in subsequent studies. Upon further investigation of the impact of mutations on codon translation, we observed a distinct pattern in the codon mutation map of the vaginal genomes, with most mutations occurring along the opposite diagonal (Fig. S1, Table S3). The location and quantity of SNVs within genes may directly influence gene function and consequently shape microbiome evolution.

### Comparison of enriched metabolic pathways caused by genetic mutations

After demonstrating the co-evolution of the taxonomy and mutational signature of the vaginal microbiota among individuals with CC, we further investigated alterations in microbial functionality. To perform enrichment analysis of functional pathways, we selected the top 500 genes with the highest number of SNVs across all groups and compared them using the KEGG.

Our findings revealed limited similarity in terms of functional pathways among the microbiomes in these four groups, indicating significant changes in microbial function during CC progression (Table S4). Interestingly, only two functional pathways overlapped between these groups: genetic information processing protein family (B09182) and pyruvate metabolism (00620).

**Fig. 4:**
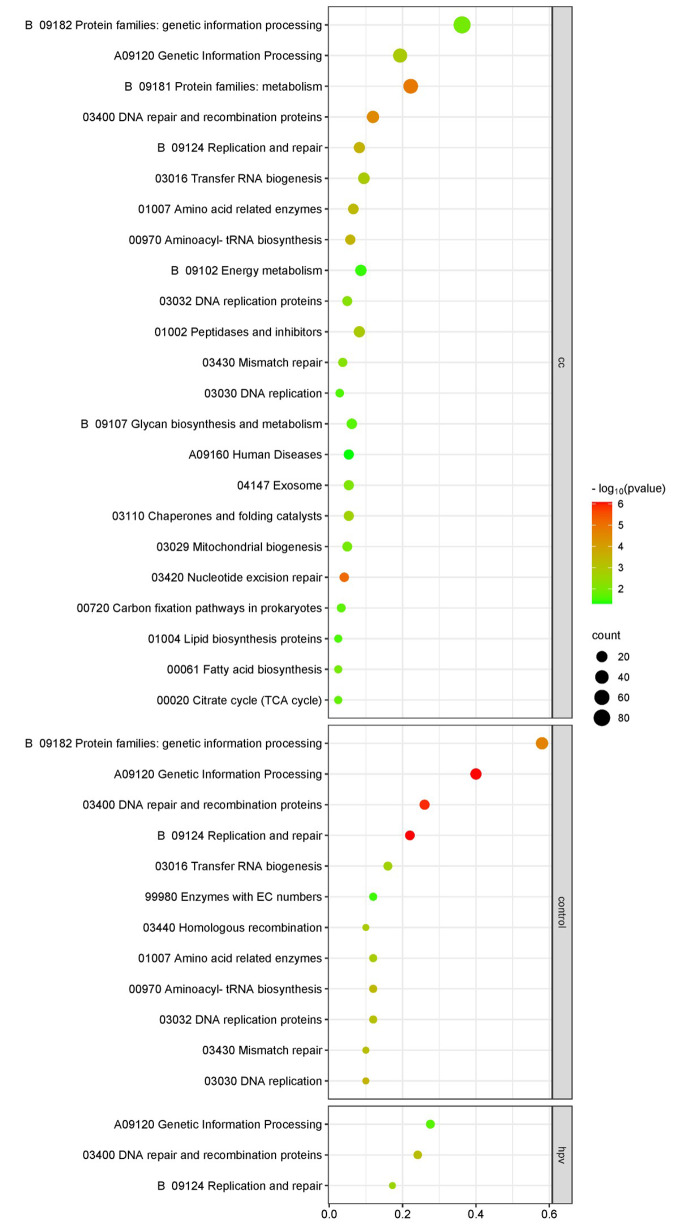
Enrichment pathways of mutated genes in CC, CIN, HPVi patients and controls

Further analysis of the enriched metabolic pathways within each group revealed specific microbial functional changes at different stages of CC progression (Fig. 4). The vaginal microbiota of patients with HPVi exhibited active nucleotide biosynthesis, including DNA replication and recombination proteins, transfer RNA biogenesis, and mitochondrial biogenesis. In contrast, patients with CC demonstrated a significantly higher abundance of signaling and cellular-associated protein families. However, the CIN group displayed fewer enriched functional pathways while having a higher count in the gene information processing pathway. Nevertheless, the healthy controls showed high levels of enrichment in sugar metabolism, including pyruvate, starch, and sucrose metabolism. These findings indicate distinct functional features between the vaginal microbiomes of patients with dysbiosis and healthy women.

### Multimodal microbial marker in the construction of CC diagnostic models

The performance of diagnostic models is highly dependent on feature selection. Therefore, we employed four distinct biomarker types to ascertain the optimal features for constructing our models: vaginal microbiome abundance, the number of SNVs within contigs, the number of SNVs in genes, and SNV abundance. Moreover, we used the random forest algorithm to train four separate diagnostic models that effectively distinguished patients with CC, CIN, and HPVi from healthy controls, with each model based on a single feature.

**Fig. 5:**
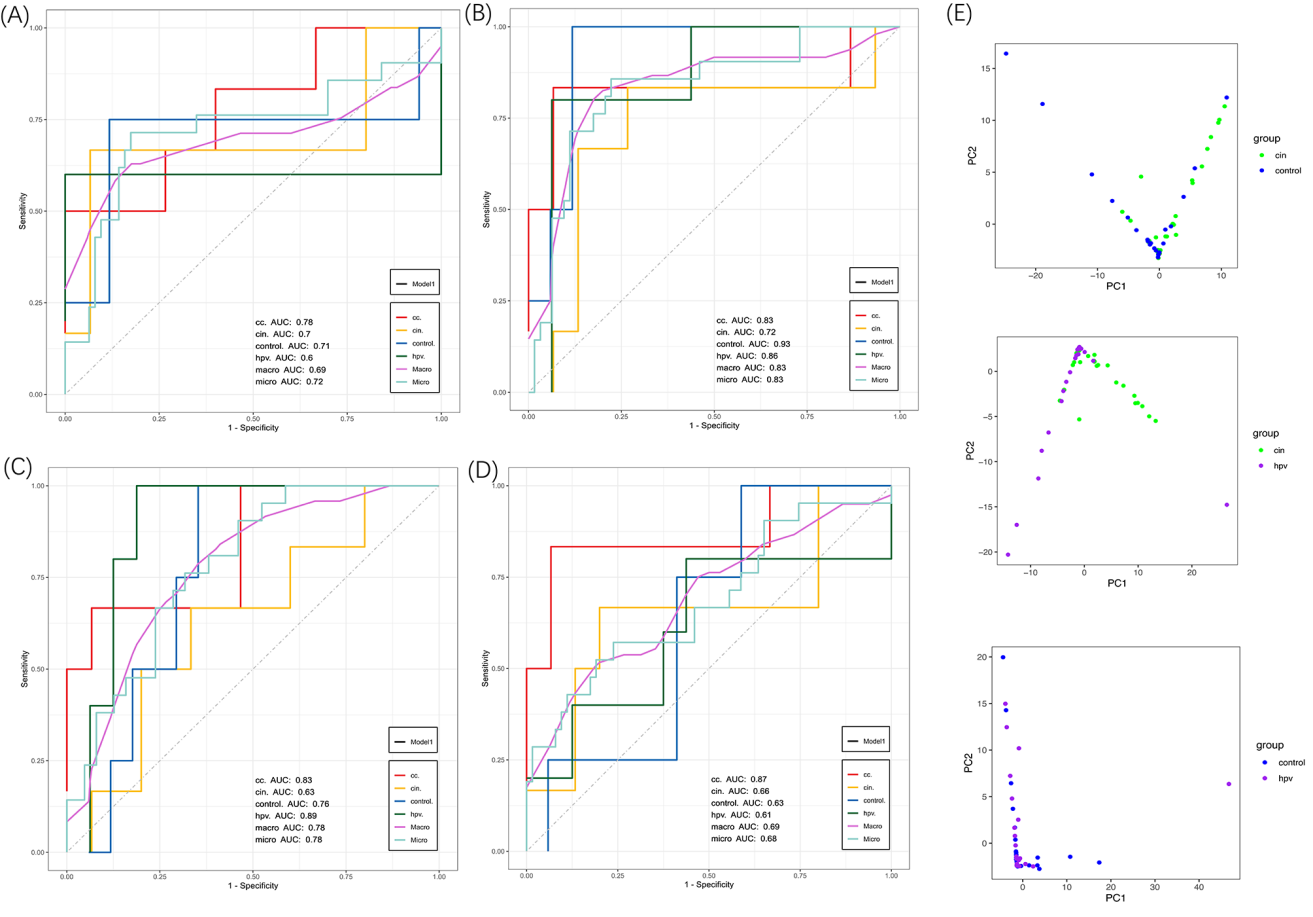
Diagnostic models based on microbial monomodal biomarkers. (**A-D**) Receiver operating characteristic (ROC) curves and area under the curve (AUC) for the 4 individual feature models. Intestinal species abundance (**A**), number of SNVs within contigs (**B**), number of SNVs within genes (**C**), and SNV abundance alone (**D**) were used as features of the model. (**E**) PCA plot of pairwise comparison of the biomarker in groups, testing the ability of biomarkers to separate the two groups

**Fig. 6:**
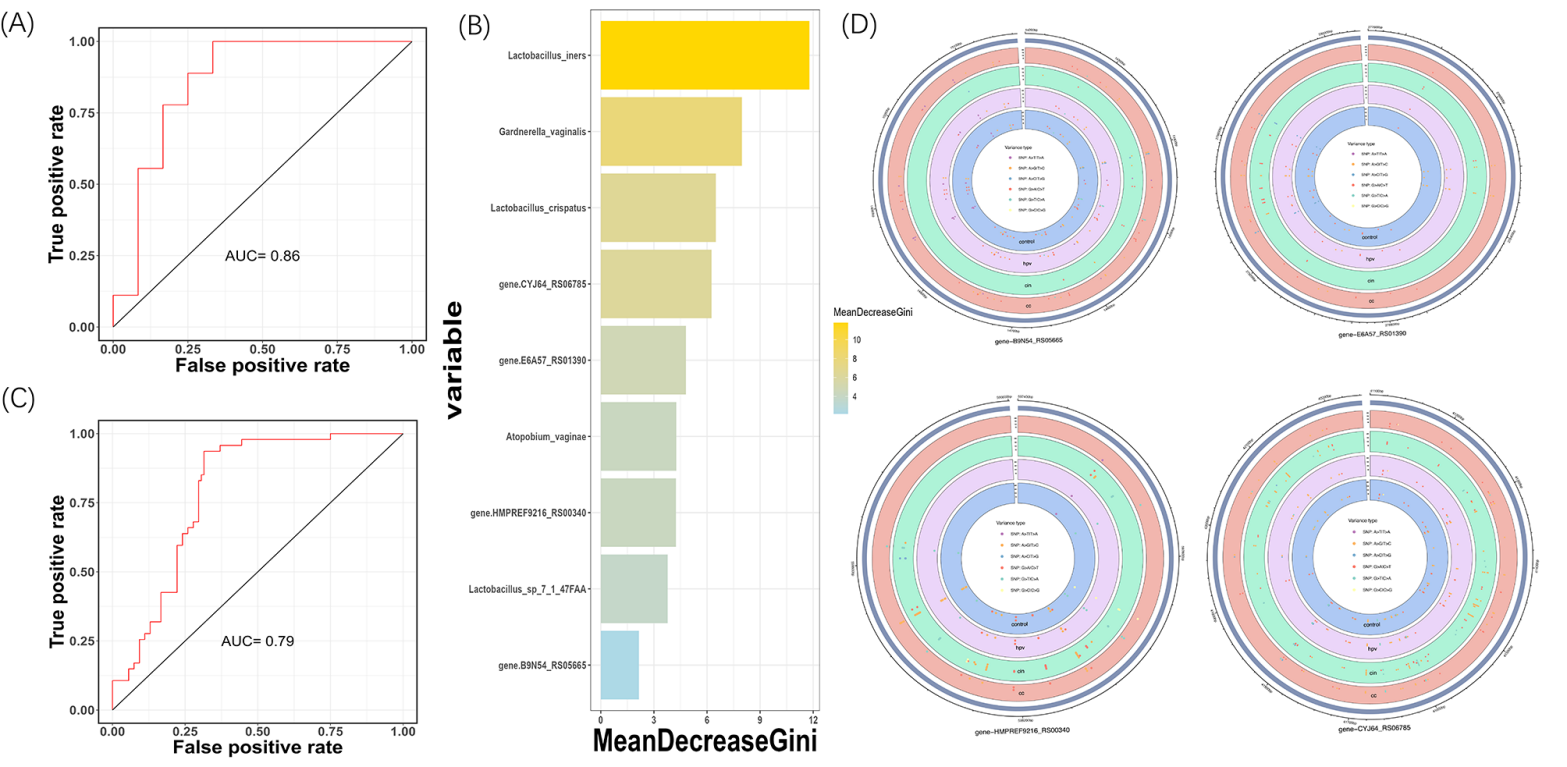
Diagnostic models based on microbial multimodal biomarkers. (**A**) ROC curves and AUC for the combined feature model in internal discovery set. (**B**) The 9 features in 2 types that were combined to build the final disease diagnosis model and ranked according to their contribution to the random forest model. (**C**) ROC curves and AUC for the combined feature model in external testing set. (**D**) Mutation types and positions of SNV within the gene in gene-B9N54_RS05665,gene-E6A57_RS01390, gene-HMPREF9216_RS00340 and gene-CYJ64_RS06785, the horizontal coordinate represents the sample name, the vertical coordinate represents the site

The important scores of the different features were obtained and ranked for further screening of biomarkers (Fig. S2). We found that the three diagnostic models (cc.AUC = 0.83; cc.AUC = 0.83; cc.AUC = 0.87) based on SNV number were superior to the general model of species abundance (cc.AUC = 0.78), further underlining the sensitivity of vaginal microbial SNV biomarkers (Fig. 5A-D). Considering their clinical applicability in terms of efficiency and cost-effectiveness, based on the RFE result and important score of 4 models, we further identified a minimal set of biomarkers consisting of 5 species and 4 genes as the input of our final diagnosis model (Fig. 6B).

To further validate the discriminatory potential of SNVs in distinguishing between HPVi, CIN, CC, and healthy controls, we performed principal component analysis (PCA) to visualize the separation between these groups. Based on SNVs in genes, our analysis revealed that vaginal microbiota highly differed between HPVi, CIN and healthy controls in pairwise comparison (Fig. 5E).

To investigate whether the integration of multimodal biomarkers can enhance predictive capabilities, we proposed a final binary classification model to distinguish tumor (CC or CIN) and non-tumor (HPV or healthy) using the minimal set of biomarkers combination identified above. Notably, an enhanced performance (AUC = 0.86) was observed in the internal discovery cohort, with an acceptable performance (AUC = 0.79) in the external testing set. (Fig. 6A-C). Consistent with previous studies, the *Lactobacillus* genus emerged as highly significant in our model, including the most influential biomarker, *Lactobacillus iners*, along with two other species.

To further explore the relationship between biomarkers and diseases, we analyzed the functions of the four genes selected in our final model. The mutation types and locations of SNVs within the gene are shown in the figure (Fig. 6D), where different colored dots represent distinct mutation types, and their positions indicate the genomic locations of these mutations. To gain insights into the enriched SNV functions in CC, we elucidated their respective functions using feature tables obtained from a public database. The concentric rings in our study represented different disease types, with each ring corresponding to a specific group. The innermost to outermost rings represent the control, HPVi, CIN, and CC groups, respectively.

The B9N54_RS05665 gene, located in NZ_NDYD01000021.1, in *Finegoldia magna*, is implicated in DNA methylation modifications. The CYJ64_RS06785 gene, functionally annotated as inositol-3-phosphate synthase, belongs to the NZ_PKJK01000002.1, family of *Gardnerella vaginalis*. The presence of HMPREF9216_RS00340 and E6A57_RS01390 in *Lactobacillus iners* and *Lactobacillus crispatus* suggests an evolutionary association with changes in cervical conditions. HMPREF9216_RS00340 encodes the NUDIX hydrolase, which plays an early role in the pterin branch of the folate synthesis pathway [22, 32]. The E6A57_RS01390 gene is associated with the transcription-repair coupling factor, facilitating access to repair proteins in lesions. We observed that the relative abundances of *Finegoldia magna*, *Gardnerella vaginalis* and two *Lactobacillus* spp. varied significantly among the four groups, which highlights the validity of the gut microbial SNV biomarkers from another point of view.

## Discussion

While numerous reports have focused on the taxonomic involvement of the microbiota in the pathogenesis of CC, there has been significant neglect in exploring the evolutionary dynamics at the genetic level within microbial species [11, 33–35]. Previous studies on other cancers have demonstrated that the host disease status is more strongly associated with microbial gene signals than with microbial species, highlighting the importance of gene-level analysis [36]. In this study, we conducted a comprehensive investigation at the taxonomic, functional, and SNV levels to elucidate the presence of multimodal signatures within vaginal microbial species with the aim of gaining insights into their potential role in CC.

When exploring CC-associated microbial taxonomic signatures, consistent with previous studies, we observed that the depletion of the genus *Lactobacillus* and increased anaerobic genera were significantly associated with the progression of CC. Our findings are in accordance with recent studies indicating that bacterial vaginosis (BV) is linked to adverse gynecological and obstetric outcomes, including an increased risk of sexually transmitted infections and cancer [35, 37].

After confirming the compositional differences in the microbiomes of CC, CIN, HPVi, and healthy controls, we further investigated their microbial genomic variation profiles. SNVs were extracted and annotated for the analysis of metagenomic data, and we found that the frequency of nonsense mutations was much lower than that of missense and silent mutations in all groups. Compared with nonsense mutations, missense and silent mutations have relatively small impact on the structure and function of the encoded proteins. As previously defined as ‘dark SNVs’, these variants may have diagnostic potential despite the absence of alterations in encoded amino acids [38]. Also, all types of mutation occurred more frequently in CIN group though not significantly when compared to the CC group. This suggests a different evolutionary strategy of the microbiome during lesion progression. Across the four groups we observed that most genes with higher mutation frequencies also underwent multiple types of mutations, including silent and missense mutations. Unsurprisingly, the top 30 genes with the highest mutation frequencies mainly belonged to BV or dysbiosis-associated community (CST IV), including *Lactobacillus spps.*, *Streptococcus agalactiae* and *Gardnerella vaginalis*. Among the 30 genes, *Gardnerella vaginalis* harbored 17 of them, which exhibit a relatively high mutation rate in the tumor-associated group(CC and CIN). This finding once again underscores its significant role during vaginal dysbiosis and suggests that microbial genetic evolution may occur concomitantly or even precede oncogenic transformation. Interestingly, although only one gene (gene-AMM49_RS05120) was derived from *Streptococcus agalactiae*, it showed the highest mutation frequency, encoding tetracycline resistance ribosomal protection protein Tet(M). Multiple studies have indicated that upregulation of Tet(M) in *Streptococcus agalaciae* mediate its resistance to tetracycline and its derivatives, which might also associated with cervical carcinogenesis [39, 40].

Additionally, we identified two microbial mutational signatures associated with cancer transition. Signatures A and B were both characterized by a predominance of C > T substitutions but differed mainly in the proportion of C > A, C > G, and T > G substitutions. The presence of signature B in CC groups suggests that the microbiome may undergo “driver” mutations with the development of precancerous lesions, which persist throughout cancer progression. Although mapping of the bacterial 96-mutational signature is still lacking, we compared it with the mutational signatures observed in human cancers. Interestingly, our defined signature A corresponds to Signature 1 A and our defined signature B corresponds to Signature 1B found in human cancers [31]. Furthermore, CC was shown to exhibit Signature 1B but lacks Signature 1 A. However, further studies are required to confirm the link between microbial and cancer signatures.

The enriched metabolic pathways in the cervicovaginal microbiota further demonstrate differential evolution in the microbiome. Generally, the microbiome in cervical disease exhibits active nucleotide biosynthesis. Active genetic information and signaling processes (including DNA replication, mitochondrial biogenesis, and tRNA biosynthesis), which are involved in a range of microbial activities, may indicate their survival strength. The functions of differentially expressed genes in healthy control microorganisms were mainly concentrated in sugar metabolism. These features partially reflect the ability of protection by predominant *Lactobacillus* in the female genital tract from bacterial pathogens. Specifically, antimicrobial metabolites produced by *Lactobacillus crispatus* have been shown to decrease glucose levels and the production of phenyl lactate and N-acetylated amino acids in epithelial cervical cell models [37].

In clinical practice, the diagnosis of patients with CIN and CC typically involves a thin-prep cytology test, colposcopy, and biopsy. However, due to the inconvenience associated with these current diagnostic tools, there is an urgent need for rapid and noninvasive diagnostic methods. In this study, we hypothesized that composition and gene signatures could serve as reliable biomarkers for predicting CIN and CC in patients. Thus, we constructed four diagnostic prediction models based on microbiome signatures: vaginal species abundance, number of SNVs within contigs or genes alone, as well as SNV abundance alone. Among these models, those incorporating SNVs were found to be superior to the general model based on species abundance alone. This highlights the sensitivity of SNVs as potential biomarkers.

Also, considering the efficiency and cost effectiveness in clinical practice, we developed an integrated diagnostic model that combines minimal set of biomarkers, including species abundance and SNVs in gene. The ROC curve analysis demonstrated that microbial biomarkers effectively distinguished tumor group and non-tumor group both in the internal discovery cohort and external testing set. Although some studies have already used the vaginal microbiome as a diagnostic biomarker, they have focused solely on taxonomic unit [35]. One remarkable finding in our study is the diagnostic model constructed with SNV biomarkers achieved higher AUC than species abundance. Therefore, compared to previous studies, our analysis further considered the complexity of microbiome-host interactions and highlight the potential for this cost-effective noninvasive biomarker panel to serve as an invaluable tool for early screening of CC.

Consistent with previous studies, we considered that *Lactobacillus spp.* can be used as good biomarkers for the distinction [35], including *Lactobacillus iners* and *Lactobacillus crispatus*. Specifically, unlike other *Lactobacillus* species that inhibit virus infection, *L. iners* is considered a transitional species in a dysbiotic state. It can produce inerolysin and L-lactic acid, which are associated with viral infections [41]. Recently, *Lactobacillus iners* has emerged as a significant predictor of non-response to chemoradiation for cervical cancer and is associated with reduced recurrence-free survival [42]. Other pathogenic bacteria, especially anaerobes, such as *Gardnerella vaginalis* and *Atopobium vaginae*, also serve as powerful biomarkers for the prediction of cervical lesions [35, 43, 44].

Furthermore, the screened gene signatures with the highest number of SNVs were associated with DNA modification, transcriptional repair, cellular signaling, and folate synthesis. Nudix hydrolase belonging to *Lactobacillus iners* has been reported to be associated with trimethoprim-sulfamethoxazole resistance in the gut microbiome, which depyrophosphorylates dihydroneopterin triphosphate in the pterin branch of the folate synthesis pathway [45]. Similarly, studies have demonstrated that *Lactobacillus iners* is an obligate producer of L-lactate, which has been associated with elevated levels of L-lactate in the cervical tumor microenvironment and subsequent modulation of various metabolic pathways within tumors [42]. Therefore, considering the significant role of *Lactobacillus iners* during cervical disorders, we postulate that its folate synthesis pathway may represent an unexplored signaling cascade influencing tumor metabolism, thus presenting potential therapeutic targets.

Overall, this study provides compelling evidence supporting the potential of SNVs in early recognition and screening of cervical cancer, as well as interventions targeting *Lactobacillus iners* within the tumor microenvironment for future clinical translation. However, this study has some limitations. First, the vaginal microbial samples may be regionally and ethnically heterogeneous, and only Chinese subjects were included in our study. Secondly, the discovery and validation cohorts in our study were characterized by a relatively modest sample size. Before widely integrated into current clinical workflows, larger multicenter verifications are necessary to facilitate a more accurate analysis of the correlation between the genetic co-evolution of the microbiome and different cervical statuses. Additionally, the comprehensive SNV-based diagnostic model requires further validation in cohorts.

## Conclusion

The vaginal microbiome exhibits distinct SNV evolution along with the severity of cervical lesions. Moreover, SNV-associated multimodal biomarkers demonstrated remarkable discriminatory capability across the four different cervical statuses, particularly for the accurate identification of CC. Further clinical validation is warranted to elucidate the underlying biological mechanisms of SNV evolution in CC progression and to evaluate the performance of a comprehensive diagnostic model.

### Electronic supplementary material

Below is the link to the electronic supplementary material.

Supplementary Material 1

Supplementary Material 2

Supplementary Material 3

## Data Availability

All data from the metagenomics sequence can be obtained from NCBI Sequence Read Archive database (accession number: PRJNA771720, PRJNA1057216, PRJNA779415).

## Abbreviations

AUC: Area under the curve
BV: Bacterial vaginosis
CC: Cervical cancer
CIN: Cervical intraepithelial neoplasia
HPV: Human papilloma virus
HPVi: Human papilloma virus infection
KEGG: Kyoto Encyclopedia of Genes and Genomes
PCA: Principal component analysis
RFE: Recursive feature elimination
ROC: Receiver operating characteristic
SNV: Single nucleotide variant
