# Supplementary figures and images for: Co-evolution of vaginal microbiome and cervical cancer
Source: J Transl Med. 2024 Jun 11;22:559. doi: 10.1186/s12967-024-05265-w (PMC11167889; doi:10.1186/s12967-024-05265-w)

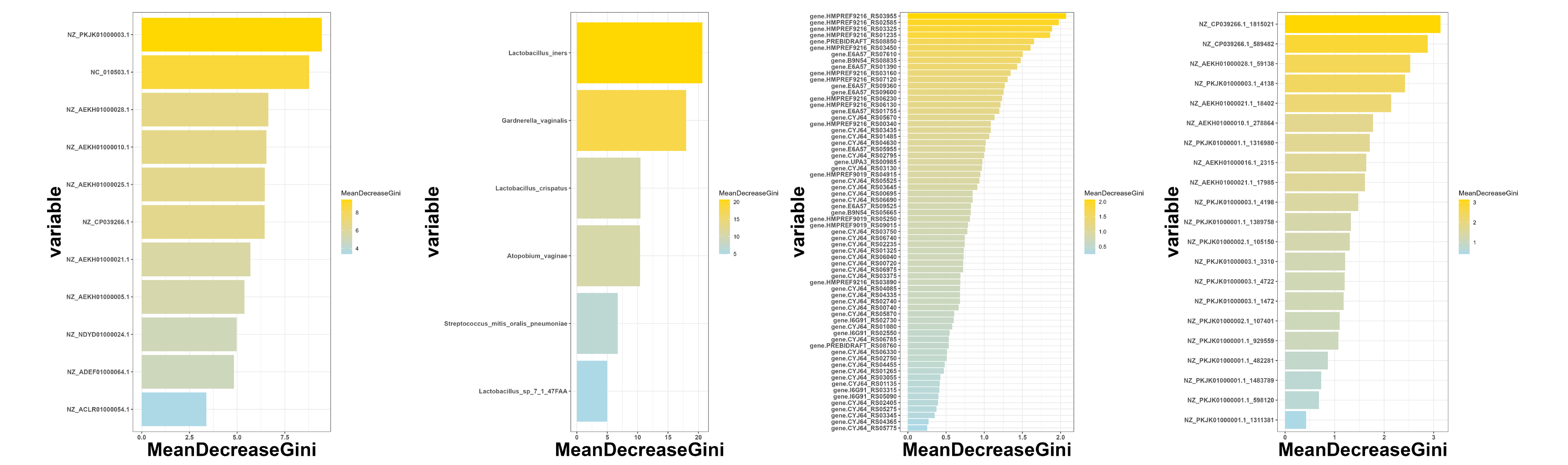

Supplement: Supplementary file 3 — Supplementary Material 3 [file 12967_2024_5265_MOESM3_ESM.jpg]
